# On Dispersion Compensation for GAW-Based Structural Health Monitoring

**DOI:** 10.3390/s23094282

**Published:** 2023-04-26

**Authors:** Alexander Backer, Viktor Fairuschin, Klaus Stefan Drese

**Affiliations:** Institute of Sensor and Actuator Technology, Coburg University of Applied Sciences and Arts, Am Hofbräuhaus 1b, 96450 Coburg, Germanyklaus.drese@hs-coburg.de (K.S.D.)

**Keywords:** guided acoustic waves, Lamb waves, structural health monitoring, total focusing method, signal coherence factor, dispersion compensation

## Abstract

Guided acoustic waves (GAW) have proven to be a useful tool for structural health monitoring (SHM). However, the dispersive nature of commonly used Lamb waves compromises the spatial resolution making it difficult to detect small or weakly reflective defects. Here we demonstrate an approach that can compensate for the dispersive effects, allowing advanced algorithms to be used with significantly higher signal-to-noise ratio and spatial resolution. In this paper, the sign coherence factor (SCF) extension of the total focusing method (TFM) algorithm is used. The effectiveness is examined by numerical simulation and experimentally demonstrated by detecting weakly reflective layers with a highly dispersive A0 mode on an aluminum plate, which are not detectable without compensating for the dispersion effects.

## 1. Introduction

GAW or Lamb waves are increasingly used for large area monitoring of surfaces where other methods, such as ultrasonic probes, are not practical due to their small measuring range. Due to the property of Lamb waves to completely penetrate the substrate material, it is also possible to monitor areas to which there is no direct access [1,2].

For the identification of defects or cracks in the structure to be monitored, the symmetric fundamental mode (S0) of the Lamb waves is often used [3,4]. At low frequency plate thickness product, the S0 mode is almost non-dispersive, thus facilitating data evaluation. Various imaging techniques have been developed to evaluate the data often measured using piezoceramics [5,6,7,8]. Two of the best known are the synthetic aperture focusing technique (SAFT) [1,9,10,11,12,13] and the TFM [5,14,15,16,17]. These can be used to combine data recorded by different transmitters and receivers to produce an image of the surface being monitored. Knowing the propagation speed of the waves and the position of the transmitters and receivers, the measured signals can be shifted in relation to each other in such a way that the signals reflected at a defect are constructively superimposed and these signals are thus amplified and become visible.

In addition to the comparatively easy-to-use SAFT and TFM algorithms, there are also very powerful beamforming algorithms such as delay multiply and sum (DMAS) [18]. However, DMAS is much more computationally intensive and requires a constant phase position due to the multiplication of the signals. This is not given by the dispersive property of Lamb waves. However, the method used in this paper for TFM could also be applied to DMAS.

Various extensions of the basic SAFT and TFM algorithms have been developed to increase the spatial resolution or to detect weakly reflecting defects. The aim is usually a higher signal-to-noise ratio (SNR) and a higher spatial resolution. Among others, cross correlation [19] and autocorrelation [20] are used.

Other methods use a noise signal [21] or diffuse sound fields [22]. However, the dispersive nature of Lamb waves and the related signal stretching leads to reduced spatial resolution. This can be counteracted by removing the dispersion effects in the data preprocessing [23,24,25,26,27,28,29,30,31]. Depending on the methods used, however, the signals are shifted in phase or frequency [32,33,34,35]. If a stable phase position is required for subsequent data processing, a method must be selected in which phase stability is ensured.

There are also approaches to increase the speed of the TFM [36]. Other methods try to monitor surfaces with as few as possible piezos distributed over a large area [16,37,38].

The latest algorithms attempt aim to both to increase the measurement speed and to reduce the influence of the transmitted signal in the case of defects located very close to the phased array [39]. There is also great potential in taking into account the phase of the received Lamb waves [19,40].

The sign coherence factor (SCF) or the phase coherence factor (PCF) are extensions that take the phase into account in addition to the amplitude [41,42]. However, the use of these extensions requires that the phase is also the same for signal propagation paths of different lengths. For Lamb waves, this is approximately true for the S0 mode, provided it is excited at low frequency plate thickness product. With the property of being particularly sensitive to changes inside the structure to be monitored, S0 modes are especially well suited for detecting cracks or material failure and are therefore used, for example, in aircraft construction [1]. A disadvantage of the S0 mode, however, is the lower sensitivity to changes on the surface of the wave guiding material. In contrast to the S0 mode, the A0 mode is particularly sensitive to the surface [4,43]. Because of the strong dispersive property of the A0-mode, extensions like the SCF or PCF can not be applied readily. However, by compensating for the dispersion effects and the phase shift caused by them, it is possible to use the A0 mode and, with the help of the extended algorithms, to detect even very weakly reflecting defects and to localize them precisely.

In this paper, the necessary steps are described and the effectiveness of the method is confirmed using simulated data. The verification in the experiment is carried out by detecting artificially applied weakly reflective flaws on the surface of an aluminium plate, which would not be detectable or only with difficulty without compensating for the dispersion effects. A similar approach was already investigated with a 1D line array and a different piezo geometry. A 6-fold higher excitation voltage and a 16-fold signal averaging were used to increase the signal-to-noise ratio. However, this increases the demands on the transmitter electronics and extends the measurement duration by a factor of 16 [44].

At this point, it should be mentioned that other methods have already been developed to deal with the dispersive nature of Lamb waves and thereby increase the resolution of imaging techniques. One of them is Matching Pursuit. A disadvantage is the very high computational effort to generate the dictionary [31]. Moreover, the method works well only when the emitted waveform is known exactly [29]. However, due to manufacturing tolerances, the influence of the adhesive layer, and the inherent resonances of the piezos, signal distortions occur, which cause the actual transmission signal to deviate from the ideal assumed transmission signal. In addition to the lower computational effort, another advantage of the methods presented here is their robustness to these influencing factors, provided that the individual piezos of the phased array are uniformly influenced.

## 2. Materials and Methods

In this section we introduce the used TFM algorithm and the SCF extension. Furthermore, the generation of test data is described and the measurement setup for experimental verification is explained.

### 2.1. Algorithmen

#### 2.1.1. Total Focusing Method

With the TFM algorithm, the area to be monitored is divided into individual pixels. The pixel value is calculated from the measured received signals and the distance from the transmitting and receiving piezo to the respective pixel. The basic form of the TFM algorithm can be expressed by [15]

(1) $$I_{\left(x,y\right)}=\left|\sum_{i=1}^{M}\sum_{j=1}^{M}h_{i,j}\left(\frac{\sqrt{{\left(x_{i}-x\right)}^{2}+{\left(y_{i}-y\right)}^{2}}+\sqrt{{\left(x_{j}-x\right)}^{2}+{\left(y_{j}-y\right)}^{2}}}{v_{gr}}\right)\right|\;.$$

This formula can be used to calculate a single pixel value *I* with the coordinates *x* and *y*. $x_{i}$, $y_{i}$, $x_{j}$ and $y_{j}$ are the *x* and *y* coordinates of the respective transmitting and receiving piezo for a total of *M* piezos. Together with the group velocity $v_{gr}$, this results in the signal travel time of the wave from the transmitting piezo to the position (x,y) and back to the receiving piezo. For the calculation in this case not the measured signal $f_{i,j}$ but its envelope $h_{i,j}$ is used [15].

For non-dispersive or only slightly dispersive waves, the measured signal $f_{i,j}$ can also be used [36].

To monitor a complete surface, this process must be repeated for each pixel. To speed up the calculation, it is therefore advisable to calculate with matrices instead of loops.

Thus the time shift for a piezo pair is

(2) $$\tau_{i,j}=\frac{\sqrt{{\left(x_{i}-X\right)}^{2}+{\left(y_{i}-Y\right)}^{2}}+\sqrt{{\left(x_{j}-X\right)}^{2}+{\left(y_{j}-Y\right)}^{2}}}{v_{gr}}$$

where *X* and *Y* are matrices with the *x* and *y* coordinates of the pixels. The formula for calculating the complete area is therefore

(3) $$I=\left|\sum_{i=1}^{M}\sum_{j=1}^{M}h_{i,j}\left(\tau_{i,j}\right)\right|\;.$$

#### 2.1.2. Sign Coherence Factor

Various extensions have been developed to increase the effectiveness of the TFM algorithm. A particularly effective one is the SCF, which takes into account the sign of the received signals [19,41]. This is defined as

(4) $$SCF_{i}={\left|1-\sqrt{1-{\left[\frac{1}{M}\sum_{j=1}^{M}b_{i,j}\left(\tau_{i,j}\right)\right]}^{2}}\right|}^{p}$$

and is calculated separately for each transmitting piezo, where $b_{i,j}$ depends on the sign of the signal $f_{i,j}$ at the time $\tau_{i,j}$ and can be expressed by

(5) $$b_{i,j}\left(\tau_{i,j}\right)=\left\{\begin{array}{cc} -1 & f_{i,j}\left(\tau_{i,j}\right)<0\\ 1 & f_{i,j}\left(\tau_{i,j}\right)\geq 0 \end{array}\right.\;.$$

The expression under the root is comparable to the variance and can take values between 0 and 1. In the case where all signs are identical, the SCF reaches its maximum of 1. The minimum of 0, on the other hand, results when half of the signs are positive and the other half negative. How much the signs are included in the calculation can be controlled by the exponent *p*. With this method, superpositions of in-phase waves are favoured whereas areas with different phases are suppressed. A detailed study of the influence of the p-value was performed by Jorge Camacho [41]. The *p* value must not be chosen too high, as manufacturing tolerances, signal interference or artefacts caused by side lobes distort the signal. A value that is too high would ultimately lead to the suppression of weakly reflecting defects and prevent their detection. Experiments with simulated and experimentally determined data have shown that a *p* value of 2 is a good compromise between optimising the SCF algorithm and minimising falsely suppressed signal reflections. Accordingly, this value was also used for the results presented below.

Applied to the Equation (3) of the TFM algorithm, one obtains

(6) $$I_{SCF}=\sum_{i=1}^{M}\left[SCF_{i}\;\cdot \;\left(\sum_{j=1}^{M}f_{i,j}\left(\tau_{i,j}\right)\right)\right]\;.$$

Thereby, the received signal $f_{i,j}$ and not its Hilbert transform $h_{i,j}$ is used. Prior applying the SCF extension, it is important to remove the DC component from the received signal.

#### 2.1.3. Dispersion Compensation

In order to the SCF extension to be applied to dispersive Lamb waves, the dispersion effects must first be removed. In addition to the correction of the signal stretching, the restoration of the phase position is particularly important. The seven calculation steps necessary for this have been described in detail by Paul D. Wilcox [33].

A dispersion diagram is needed to apply the dispersion compensation algorithm. An aluminium plate is used for the experimental studies. With the known material properties, the dispersion diagram was calculated, for example, by solving the Rayleigh-Lamb equation [45]. With unknown material properties, the dispersion diagram can also be determined experimentally. Some approaches are based on a line measurement with a laser Doppler vibrometer [46], but this is a very expensive method. Much cheaper is the use of piezo ceramics, whereby one piezo serves as the transmitter and one or two piezos as the receiver. By evaluating the received signals, the dispersion diagrams can be calculated [47,48].

### 2.2. Generation of Test Data

The effectiveness of the method is verified in the first step by calculated test data. A point defect is assumed, at which the emitted Lamb wave is reflected. The calculations are performed in MATLAB.

For the calculation of the received signal, the distance between the emitting piezo and the defect

(7) $$s_{t}=\sqrt{{\left(x_{t}-x_{P}\right)}^{2}+{\left(y_{t}-y_{P}\right)}^{2}}$$

as well as the distance between the defect and the receiving piezo is required.

(8) $$s_{r}=\sqrt{{\left(x_{r}-x_{P}\right)}^{2}+{\left(y_{r}-y_{P}\right)}^{2}}$$

$x_{t}$, $y_{t}$, $x_{r}$ and $y_{r}$ are the *x* and *y* coordinates of the transmitting and receiving piezos. $x_{P}$ and $y_{P}$ are the *x* and *y* coordinates of the defect.

The signal reflected at the defect can be calculated using the formula

(9) $$s_{R_{t,r}}\left(t\right)=\frac{A}{\sqrt{s_{t}}\sqrt{s_{r}}}f\left(t-\Delta t_{t,r}\right)\;.$$

The reflection factor *A* determines which part of the transmitted signal f(t) is reflected at the defect. A sinusoidal burst with Hanning windowing is used as the transmission signal. During the omnidirectional propagation from the transmitting piezo to the defect, the amplitude of the Lamb wave decreases with $1\;/\;\sqrt{s_{t}}$. The impact of the Lamb wave on the defect again generates an omnidirectionally propagating wave whose amplitude decrease, on the way to the receiving piezo, is taken into account by the factor $1\;/\;\sqrt{s_{r}}$ [1]. The time shift $\Delta t_{t,r}$ represents the signal propagation time from the transmitting piezo to the defect and back to the receiving piezo. Taking into account the dispersion effects, the shape of the received signal can be calculated with the Equation (10).

(10) $$u\left(x,t\right)=\int_{-\infty }^{\infty }F\left(\omega \right)e^{i(k(\omega )x-\omega t)}d\omega$$

Here F(ω) is the fast Fourier transform (FFT) of the transmitted signal and k(ω) is the frequency-dependent wavenumber of the Lamb mode under consideration. *x* is the signal travel distance from the transmitting piezo to the defect and back to the receiving piezo and can be calculated from the Equations (7) and (8). u(x,t) can be determined by applying the inverse FFT.

(11) $$s_{t,r}=s_{t}+s_{r}$$

The resulting formula for calculating the dispersive received signal is

(12) $$s_{R_{t,r}}\left(t\right)=\frac{A}{\sqrt{s_{t}}\sqrt{s_{r}}}u\left(s_{t,r},t\right)\;.$$

### 2.3. Measurement Setup

**Figure 1:**
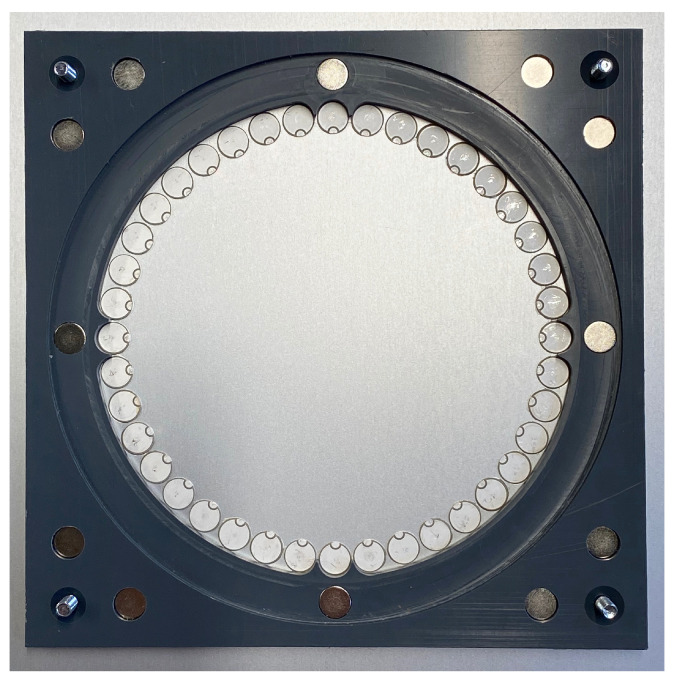
Circular-Ring array consisting of 40 disc piezos.

The experimental setup was made of a square, 1.5 mm thick aluminium plate with 1 m edge length. For the calculation, a modulus of elasticity was assumed to be 68 GPa, the density 2700 $kg/m^{3}$ and the Poisson’s ratio 0.33. At the centre of the plate was the circular ring phased array with a diameter of 110 mm as seen in Figure 1. The 40 disk piezos manufactured by PI Ceramic GmbH had a diameter of 8 mm, a thickness of 1 mm and were made of the PIC255 material. The plastic template outside the piezos was used to position and attach the electronic board whereby the individual piezos were contacted by spring contacts.

**Figure 2:**
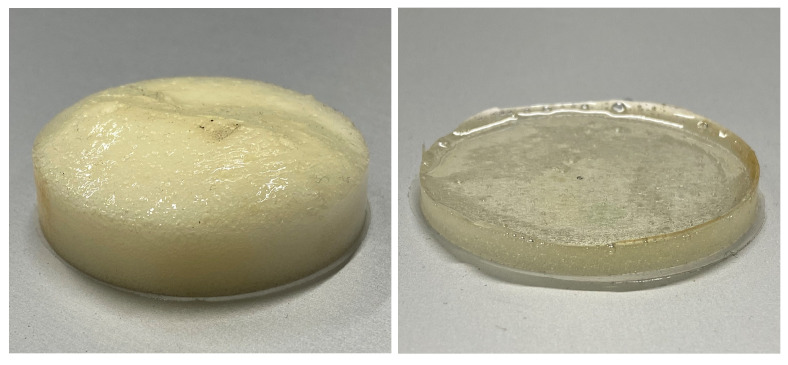
Applied defects: Flexible Polyurethane Foam (**left**) and epoxy resin (**right**).

A 20 ${}_{\mathrm{peak}-\mathrm{peak}}$, 70 kHz, 3-fold sine burst with Hanning windowing was used as the transmission signal. Transmitting and multiplexing was done by self-developed electronics and the Data acquisition by a digital storage oscilloscope (Teledyne LeCroy HDO6034). The developed electronics consists of a digital signal processor which generates the transmit signal by a digital to analog converter. By means of built-in analog multiplexers, it is possible to switch to the receive path after transmission. The signal received by the piezos is differentially amplified and forwarded to the digital storage oscilloscope. The defects to be detected are shown in Figure 2 and were circular, 46 mm in diameter and consist of cast-on flexible Polyurethane Foam (PU, FlexFoam-iT! 25) and epoxy resin (UHU Plus Schnellfest). Due to its very soft properties, the flexible Polyurethan Foam reflects only a small proportion of the Lamb wave and thus represents a weakly reflective defect. In comparison, the much harder epoxy resin reflects a larger percentage of the wave. The materials were poured into a round mold in the liquid state and cured at room temperature. The coordinates of the defects and the material used in each case are listed in Table 1.

## 3. Results

### 3.1. Simulated Data

**Figure 3:**
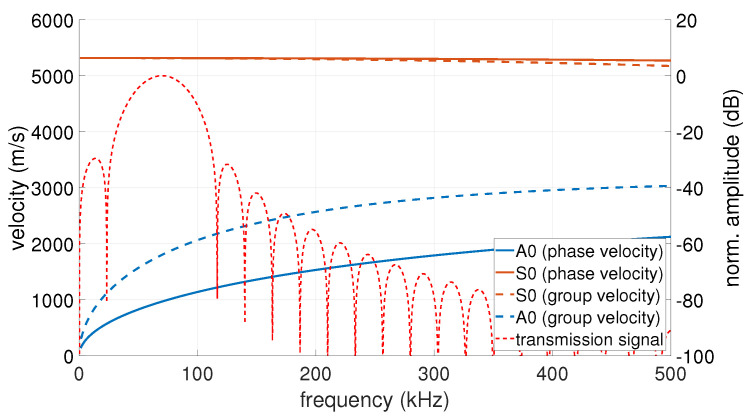
Dispersion diagram of a 1.5 mm thick aluminium plate (left axis) and the FFT spectrum of the transmission signal (right axis).

As described in Section 2.2, the effectiveness of the method was first verified on calculated test data. Data generation and data evaluation were carried out in MATLAB. As in the experiments, a circular ring phased array with 40 sensors and a diameter of 110 mm was used. The simulated transmission signal was a 3-fold sine burst with a Hanning windowing at 70 kHz. To take into account the dispersion effects, the dispersion diagram was calculated for a 1.5 mm thick aluminium plate with a modulus of elasticity of 68 GPa, a density of 2700 $kg/m^{3}$ and a Poisson’s ratio of 0.33. The mode used was the highly dispersive A0 mode at these frequencies. The dispersion diagram with the plotted FFT spectrum of the transmission signal used for the simulation and experiment can be seen in Figure 3.

Because each sensor acts both as a transmitter and a receiver, a data set of 1600 signals was created. The coordinates of the simulated defects are listed in Table 1. The positions were identical to the defects of the experimental measurements.

**Figure 4:**
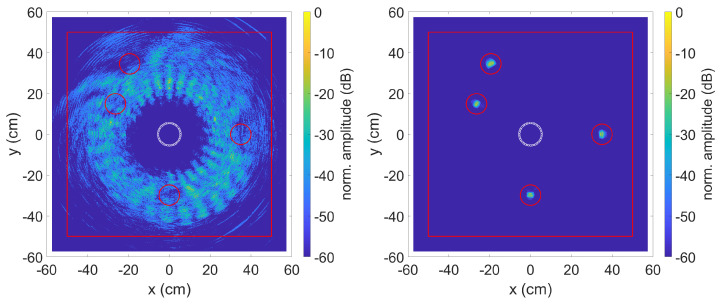
SCF TFM algorithm without (**left**) and with (**right**) dispersion compensation. Defect positions are indicated by red circles. The middle circle is the sensor position.

The data was evaluated using the TFM algorithm with SCF extension described in Section 2.1.1 and Section 2.1.2. Figure 4 shows the result on the left without and on the right with the method described in Section 2.1.3 to compensate for the dispersion effects. The red line is for comparison with the experiments and represents the edges of the aluminium plate used. The data for the plots were normalised in such a way that the highest calculated amplitude of a pixel corresponds to 0 dB.

Due to the different distances from the transmitter to the defect and back to the receiver, the strongly dispersive property of the simulated A0 mode results in a phase shift between the individual received signals. A subsequent calculation of these signals with the TFM algorithm with SCF extension leads to the result shown in Figure 4 on the left. The known positions of the simulated defects are marked by red circles with a diameter of 10 cm. An optical localisation of the defects is impossible. The right picture shows the result with applied dispersion compensation. By correcting the phase position, the SCF extension can work and the four simulated defects can be clearly recognised.

**Figure 5:**
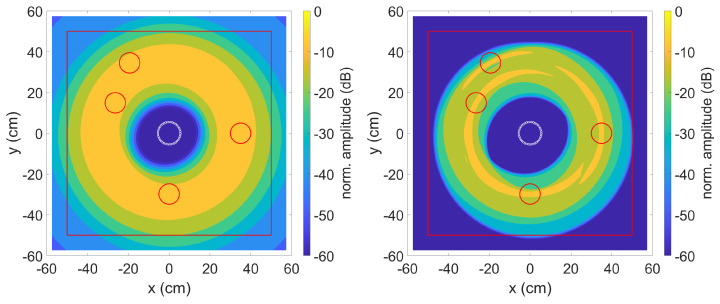
TFM algorithm with envelope signal, without (**left**) and with (**right**) dispersion compensation. Defect positions are indicated by red circles. The middle circle is the sensor position.

**Figure 6:**
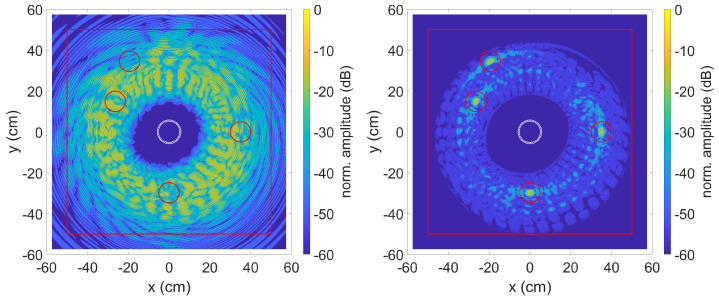
TFM algorithm without (**left**) and with (**right**) dispersion compensation. Defect positions are indicated by red circles. The middle circle is the sensor position.

In order to assess the influence of the SCF extension, Figure 5 and Figure 6 show the result of the TFM algorithm without SCF extension. In Figure 5 the envelope of the received signal was used while in Figure 6 the received signal itself was taken. On the left side without dispersion compensation and on the right side with dispersion compensation. Due to the strongly dispersive property of the used A0 mode, the signals are stretched so much that none of the flaws can be identified. If the received signal $f_{i,j}$ is used instead, the four imperfections can be identified after compensating for the dispersion effects. However, the area around the missing spots have strong amplitudes, which are mainly caused by side lobes of the phased array system. Despite compensated dispersion effects, the imperfections can only be guessed at when using the envelope of the received signal.

### 3.2. Experimental Data

In addition to the simulated data, the effectiveness of the algorithms was also tested on experimentally determined data. The experimental setup is described in Section 2.3. Before the recorded data was evaluated with the SCF TFM algorithm, it had to be filtered. For this purpose, a 10th order Butterworth bandpass with cut-off frequencies 500 Hz and 163 kHz was used. The limits were chosen so that the frequencies contained in the transmitted signal remain as unfiltered as possible. Low frequencies, such as the 50 Hz mains frequency, DC components in the signal and high-frequency signal interference were removed. Due to the low lower cut-off frequency and a relatively high filter order, the bandpass has a tendency to oscillate at low frequencies. This can be counteracted by zero padding before filtering at the beginning and end of the signal. The additional data points are removed after filtering.

**Figure 7:**
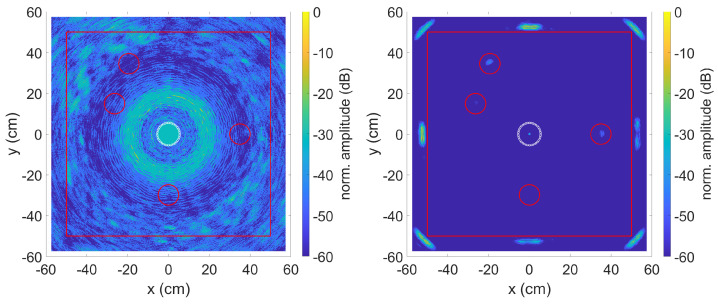
SCF TFM algorithm without (**left**) and with (**right**) dispersion compensation. Experimental data and 60 dB amplitude range. Defect positions are indicated by red circles. The middle circle is the sensor position.

As already shown with the simulated data, the 1600 individual measurement signals were evaluated with the TFM algorithm with SCF extension described in Section 2.1.1 and Section 2.1.2. Figure 7 shows the result on the left without and on the right with the method described in Section 2.1.3 to compensate for the dispersion effects. The data for the plots were normalised so that the highest calculated amplitude of a pixel corresponds to 0 dB. This is usually a reflection of the wave at the edge of the plate.

**Figure 8:**
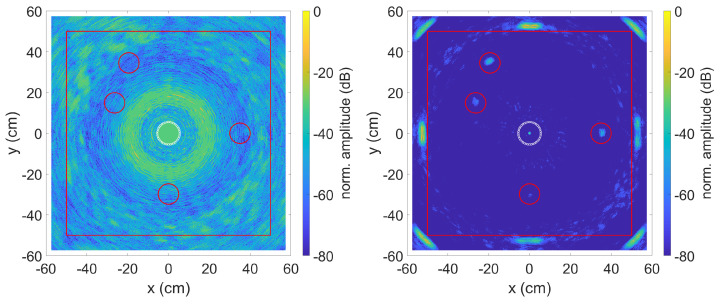
SCF TFM algorithm without (**left**) and with (**right**) dispersion compensation. Experimental data and 80 dB amplitude range. Defect positions are indicated by red circles. The middle circle is the sensor position.

As can be seen in the left image, the SCF TFM algorithm without compensation of the dispersion effects does not provide any viable results. By compensating for the dispersion effects—right image—on the other hand, both the plate edges and some defects on the plate can be recognised. With the selected data range of 60 dB, the two defects made of epoxy are clearly visible and also one of the significantly less reflective defects made of flexible Polyurethane Foam is visible. By extending the data range to 80 dB as shown in Figure 8, the second PU defect is also visible. However, this also reveals artefacts that are mainly caused by side lobes of the phased array system. The change of the data range from 60 dB to 80 dB clearly shows how strong the axis limitation affects the displayed result. The larger the data range, the more likely a false-positive detection of artefacts as defects.

At this point, it should be mentioned that it is difficult to distinguish defects from reflections at edges, weld seams or component transitions in the case of more complex geometries. One way to counteract this is to record reference signals. This can be the system under investigation at an earlier point in time or a comparable system where it is known that no flaws are present.

**Figure 9:**
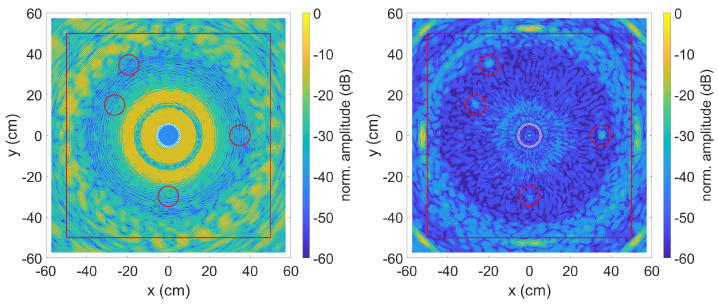
TFM algorithm without (**left**) and with (**right**) dispersion compensation. Experimental data. Defect positions are indicated by red circles. The middle circle is the sensor position.

How much the SCF extension affects the result can be seen in Figure 9. For the calculation with the standard TFM algorithm, the direct received signal was used, as it was already shown in Section 3.1 that this provides clearer results compared to the envelope. In agreement with the simulated data, the imperfections with compensated dispersion effects can also be detected here, provided that their position is known. Without knowing the position of the imperfections, they can easily be mistaken for artefacts.

## 4. Discussion

In this paper, we show a method with which even highly dispersive Lamb waves can be used for large-area surface monitoring. The crucial point here is to compensate for dispersion effects. Without this, many extensions of the imaging algorithms—such as the SCF extension for the TFM algorithm—cannot be used, as they require a constant phase position of the measured signals. Even the standard TFM algorithm using the envelope of the received signals quickly reaches its limits with dispersive signals and does not deliver evaluable results.

The effectiveness of the presented method was demonstrated by an evaluation on simulated data and was confirmed by applying it on experimental data. In the experiments, weakly reflecting flaws were applied to the surface of an aluminium plate with a thickness of 1.5 mm and measured with a phased array system consisting of 40 piezos arranged in a ring. The standard TFM algorithm was only able to detect the defects with compensated dispersion effects. Due to the low signal-to-noise ratio, however, it is only possible to identify the defect unambiguously if the position of the defect is known. Without this knowledge, various other artefacts can also be interpreted as defects. By compensating for the dispersion effects and thus also correcting the phase position, the SCF extension of the TFM algorithm could be used. With this it was possible to clearly identify all four applied defects.

Without compensating for the dispersion effects, only modes for which a weakly dispersive frequency range exists can be used. This is the case, for example, with the S0 mode and lower frequency-plate-thickness product. At the same time, long excitation signals must be used so that the bandwidth of the transmitted signal is as small as possible and thus the dispersion effects are minimised. Ultimately, this leads to a significant limitation in the modes, frequencies and excitation signals that can be used, thereby limiting the flexibility of the sensor systems. By compensating for the dispersion effects, on the other hand, the most suitable mode for the respective application can be used. For example, the S0 mode is suitable for the detection of cracks, whereas the A0 mode can be used for defects on the plate surface. This can be, for example, the detection of corrosion or the detachment or growth of a layer on the plate surface.

Furthermore, it should be stressed that a change in plate thickness or plate material does not require a change in excitation frequency for the Lamb mode to be excited again at the same frequency plate thickness product. This allows the sensors to be optimised to a specific operating frequency. At the same time, a constant excitation frequency reduces the demands on the measurement electronics and thus also the costs of these.

A further increase in sensor resolution could be achieved by increasing the reproducibility of the sensor coupling to the surface to be monitored. In addition to manufacturing tolerances in piezo production, which are difficult to influence, the adhesive bond is particularly decisive. Differences in the thickness of the adhesive layer or varying adhesive properties, e.g., due to slightly different mixing ratios of the individual adhesive components, affect the vibration behaviour of the piezos. Different signal amplitudes can be easily corrected. On the other hand, signal distortions of the transmitted signal and the resulting deviations of the phase position are difficult to correct and at the same time have an effect on the quality of the algorithms, such as the SCF extension of the TFM algorithm, which require a constant phase position.

Another possibility to increase the quality of the sensor system is the metrological determination of the dispersion diagrams. This means that the method can also be used for applications with unknown material properties or a plate thickness that cannot be determined exactly.

## 5. Conclusions

In this paper, it was successfully shown that by combining data evaluation methods, the application field and efficiency of phased array sensor systems can be greatly extended. By removing the dispersion effects, extensions such as the SCF can be used for the TFM algorithm. This can significantly increase the spatial resolution as well as the sensitivity of the sensor system. Another advantage of the SCF extension is the increase of the signal to noise ratio. Finally, the used method allows the use of dispersive Lamb waves for large area monitoring of plate-like structures and thus opens up new application areas in the field of structural health monitoring.

## Figures and Tables

**Table 1:** Positions of the defects.

Defect	Material	x (mm)	y (mm)
1	Epoxy	350	0
2	Epoxy	−194	345
3	PU	−264	149
4	PU	0	−298

## Data Availability

Not applicable.
